# Supplementary material for: An aggregon in conductin/axin2 regulates Wnt/β-catenin signaling and holds potential for cancer therapy
Source: Nat Commun. 2019 Sep 18;10:4251. doi: 10.1038/s41467-019-12203-8 (PMC6751202; doi:10.1038/s41467-019-12203-8)
Supplement: Supplementary file 1 — Supplementary Information [file 41467_2019_12203_MOESM1_ESM.pdf]

Supplementary Information

An aggregon in conductin/axin2 regulates Wnt/β-catenin signaling and holds potential for cancer therapy

Bernkopf et al.

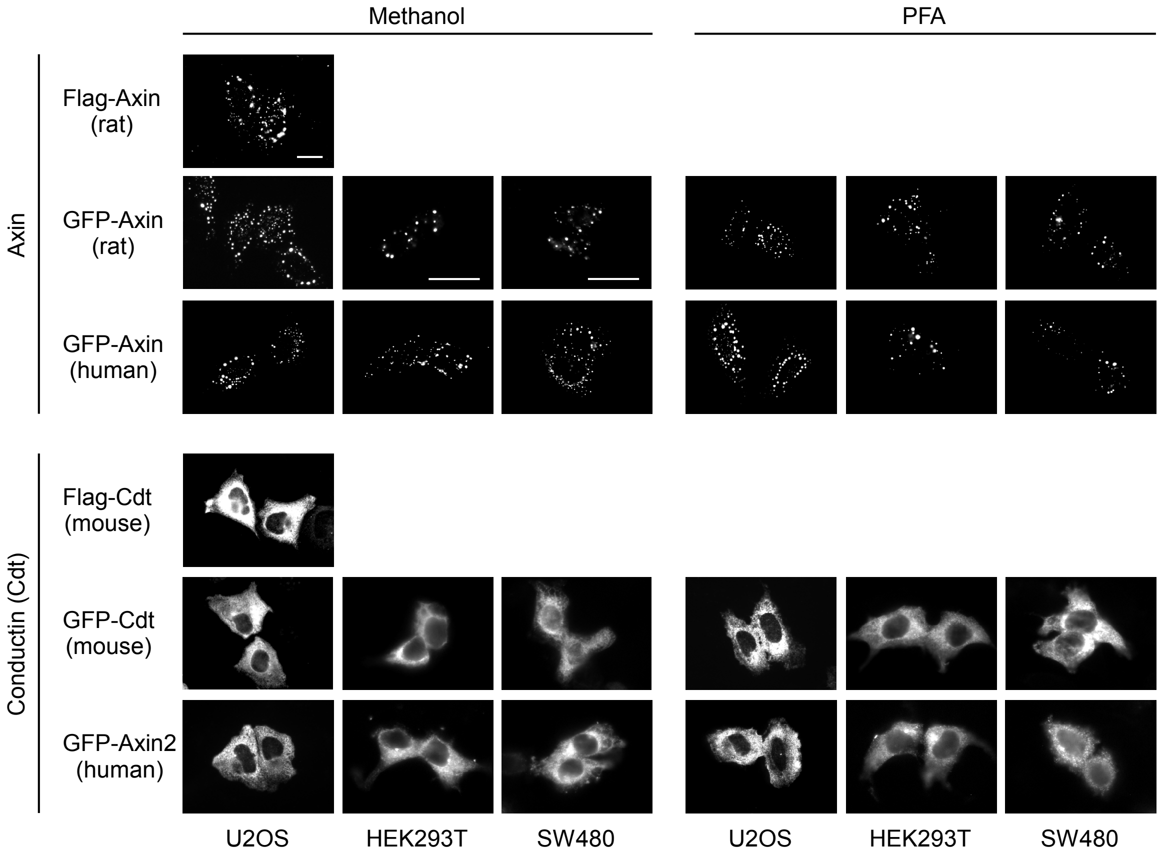

Supplementary Fig. 1 Distribution of axin and conductin. GFP fluorescence or immunofluorescence staining for Flag after methanol or PFA fixation in U2OS, HEK293T and SW480 cells transfected with indicated axin and conductin (Cdt) constructs. Scale bars: 20 µm.

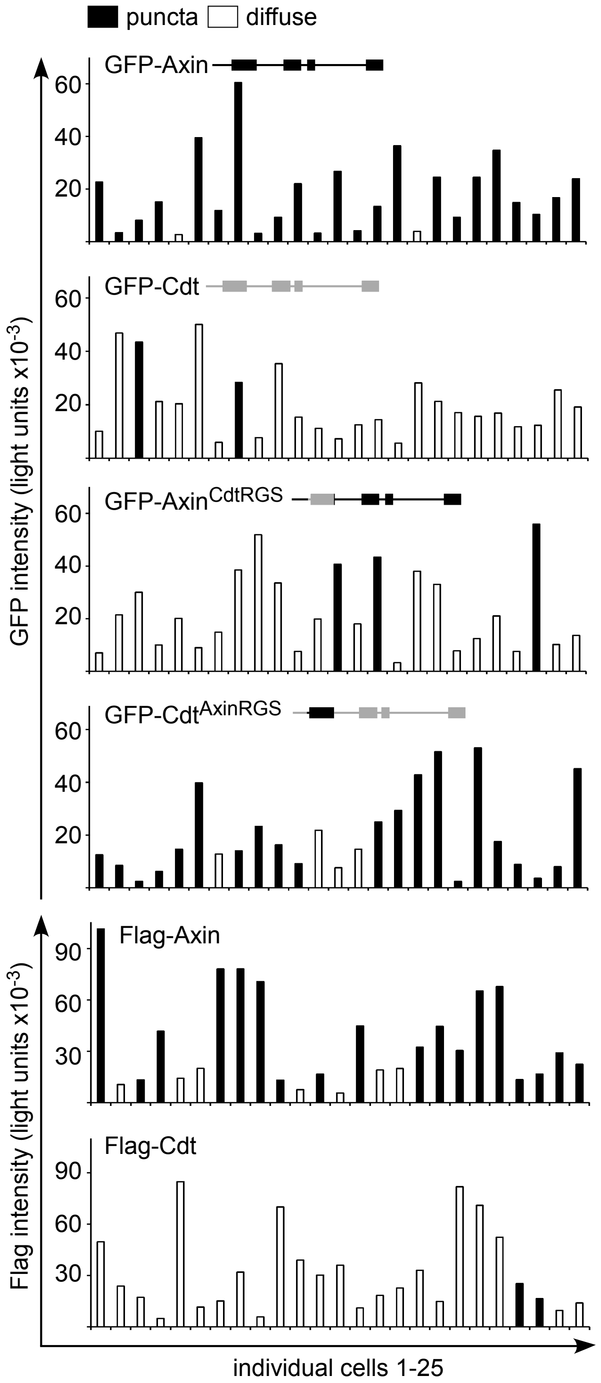

Supplementary Fig. 2 Different distribution of axin and conductin is not caused by different expression levels. Height of bars shows GFP fluorescence intensity or Flag immunofluorescence staining intensity of indicated proteins in individual U2OS cells. Color of bars shows distribution (black: puncta, white: diffuse). In the construct schemes, axin parts are shown in black, conductin parts in grey.

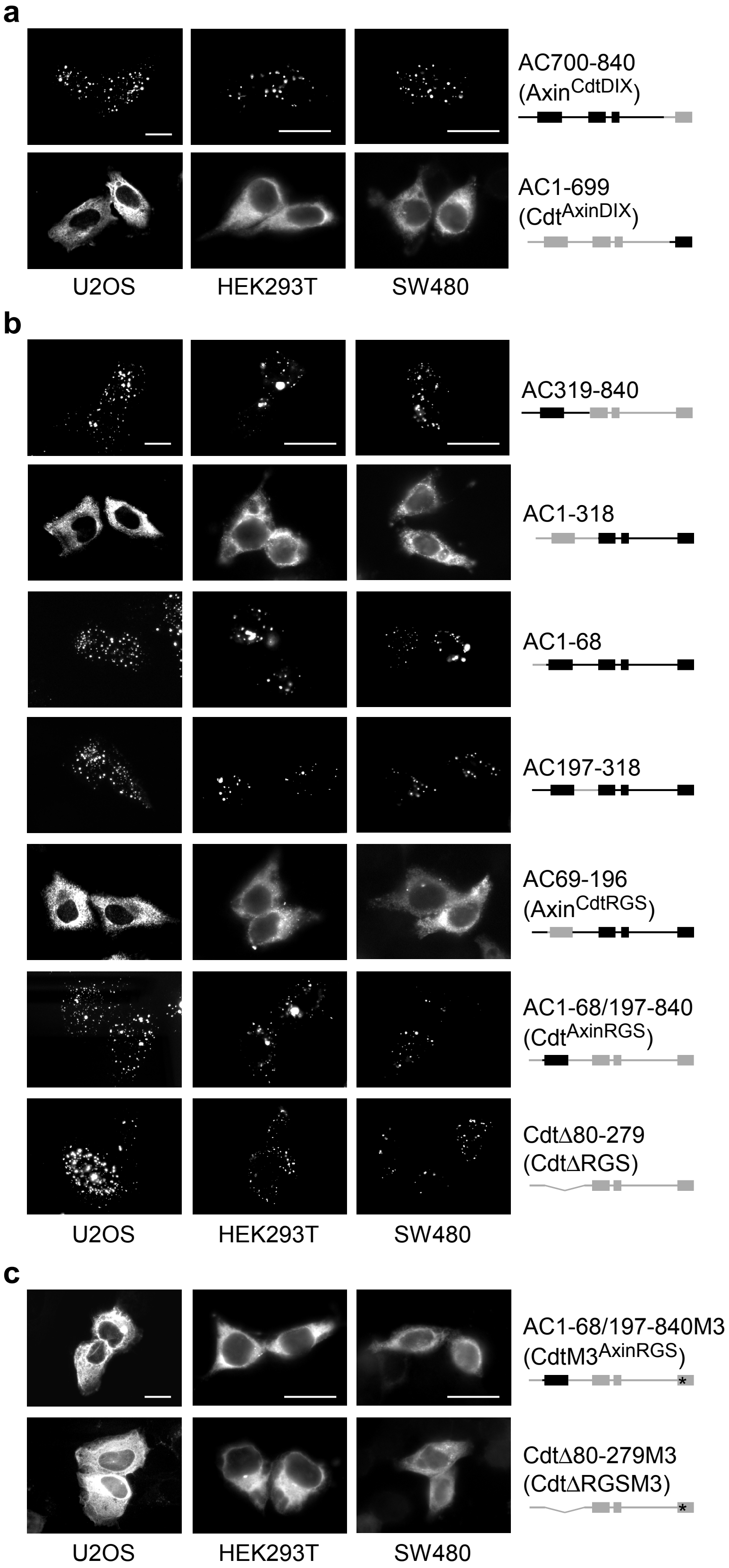

Supplementary Fig. 3 The conductin RGS domain prevents DIX-mediated polymerization. **a-c** GFP fluorescence in U2OS, HEK293T and SW480 cells transfected with GFP-tagged constructs indicated on the right. Numbers in the names of axin (A) conductin (C) chimeric constructs refer to the conductin amino acids in the construct. Axin parts in schemes are shown in black, conductin parts in grey. Scale bars: 20 µm.

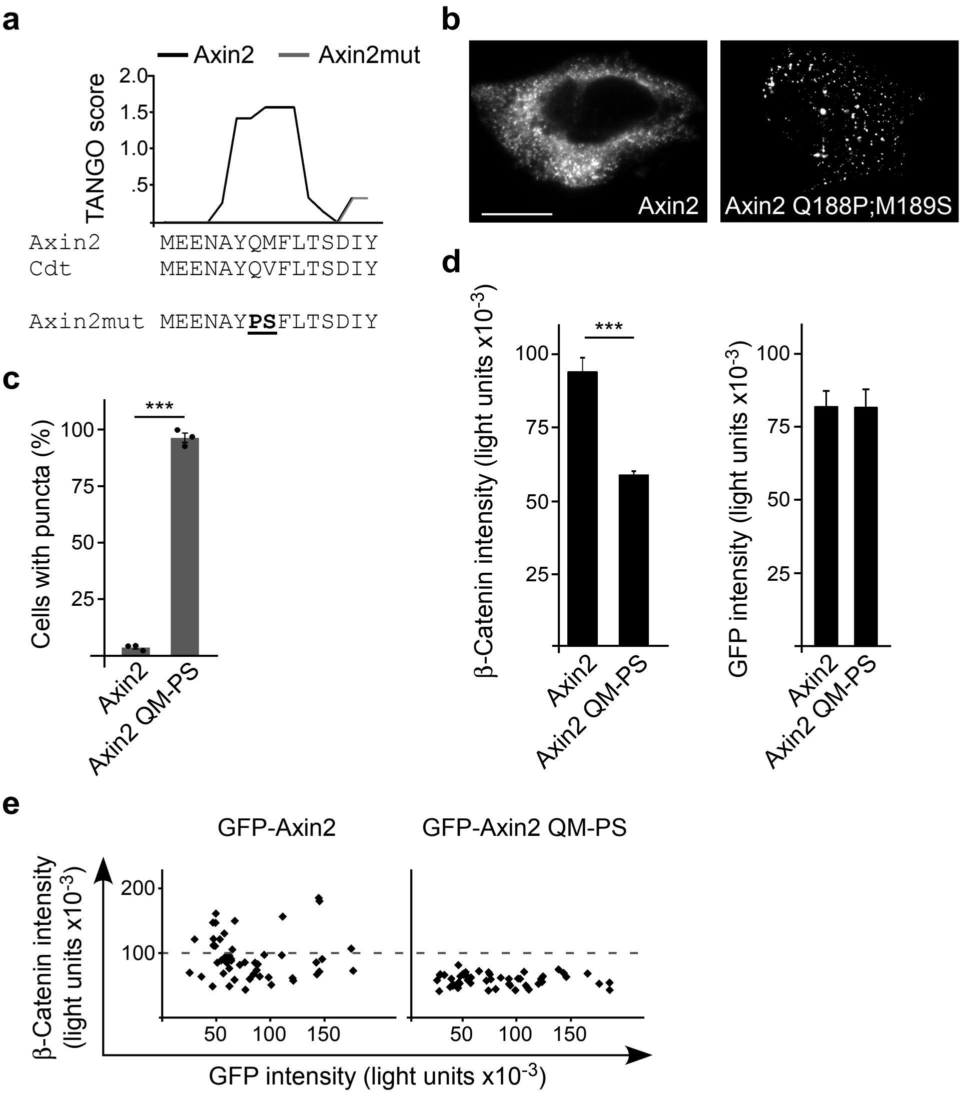

Supplementary Fig. 4 Mutation of aggregation site III in axin2 increases polymerization and β-catenin degradation. **a** TANGO aggregation propensity score for amino acids in aggregation site III of axin2 and mutated axin2 (Axin2mut)[1]. Clustal Omega alignment of axin2 and conductin sequences is shown below the diagram[2]. Mutated key residues of aggregation site III are highlighted in the sequence of mutated axin2. **b** GFP fluorescence in U2OS cells transfected with indicated GFP-tagged constructs. Scale bar: 20 µm. **c** Percentage of transfected cells showing puncta formation of indicated constructs. 1500 cells of three independent experiments as in b were analyzed. Results are mean +/- SEM (n=3). **d** Left: Quantification of β-catenin fluorescence intensities in one out of five representative experiments performed as described in figure 4a with indicated constructs. Right: Quantification of GFP intensities in analyzed cells. Results are mean +/- SEM (n=50). **e** Dot plots of β-catenin intensities plotted against the corresponding GFP intensities of individual cells analyzed in d to show distribution of the data sets. Source data are provided as a Source Data file. ***p<0.001 (Student's *t*-test).

a

```
                Axin2                       Axin

                EI          sequence        EI          sequence

Human           Q9Y2T1      MEENAYQMFLTSD   O15169      MEENTYPSFLKSD
Bonobo          A0A2R9AXL2  MEENAYQMFLTSD   A0A2R9AFS0  MEENTYPSFLKSD
Mouse           O88566      MEENAYQVFLTSD   O35625      MEENTYPSFLKSD
Rat             O70240      MEENAYQVFLTSD   O70239      MEENTYPSFLKSD
Chicken         A0A1D5PPH8  MEENAYQMFLTSD   O42400      IEDNTYPLFLKSD
Finch           H0Z192      MEENAYQMFLTSD   H0Z1M9      IEDNTYPLFLKSD
Turtle          K7G9P5      MEENAYQMFLTSD   K7FV20      IEDNTYPLFLKSD
Alligator       A0A151N2H4  MEENAYQMFLTSD   A0A151P672  IEDNTYPLFLKSD
Zebrafish       P57095      MEENAYQMFLTSD   P57094      MEENTYPLFLKSD
Salmon          A0A1S3PIU6  MEENAYQMFLTSD   A0A1S3R835  MEENTYPLFLKSD
Frog            F7ABT6      MEQEAFTSFLQSD   F6XWP7      MEDNTYPVFLKSD
                            **::*:  ** **               :*:**** *****
```

b

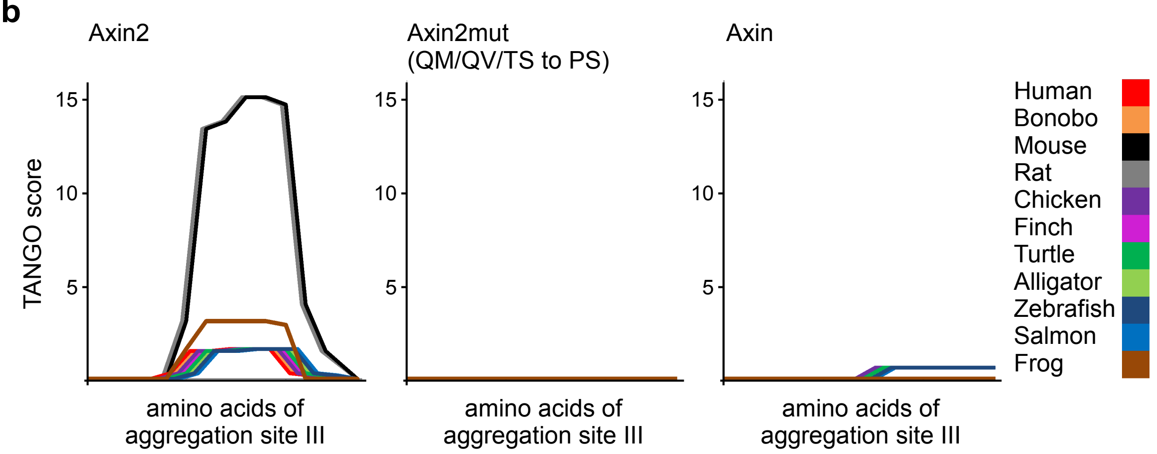

Supplementary Fig. 5 Aggregation site III is conserved among vertebrates. **a** Clustal Omega alignments at the position of aggregation site III of axin2 and axin sequences of species representing the five vertebrate classes (mammals, birds, reptiles, amphibians and fish). Identity (*) and conservation between amino acid groups of strongly similar properties (:) are indicated[2]. Mutated key residues are highlighted. EI: UniProt entry identifier. **b** TANGO aggregation propensity score of amino acids in aggregation site III of axin2 (left panel), mutated axin2 (middle panel) and axin (right panel) for indicated species[1].

Cdt	**Seq**	A	Y	Q	V	F	L
	**RSA**	21	12	46	45	8	21
		B	B	E	E	B	B

Supplementary Fig. 6 Amino acids mutated within aggregation site III are surface exposed. NetSurfP calculation of relative surface accessibility (RSA) in percent for indicated amino acids of conductin aggregation site III[3]. B: buried, E: exposed.

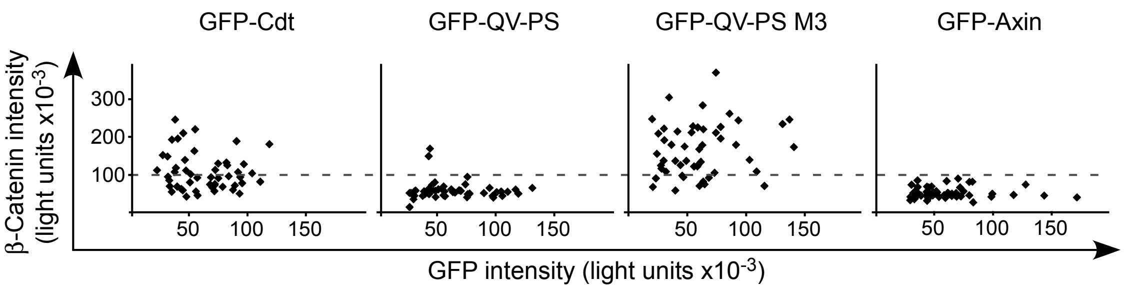

Supplementary Fig. 7 Polymerization of conductin enhances β-catenin degradation. Dot plots of β-catenin intensities plotted against the corresponding GFP intensities of individual cells analyzed in figure 4b and c to show distribution of the data sets. Source data are provided as a Source Data file.

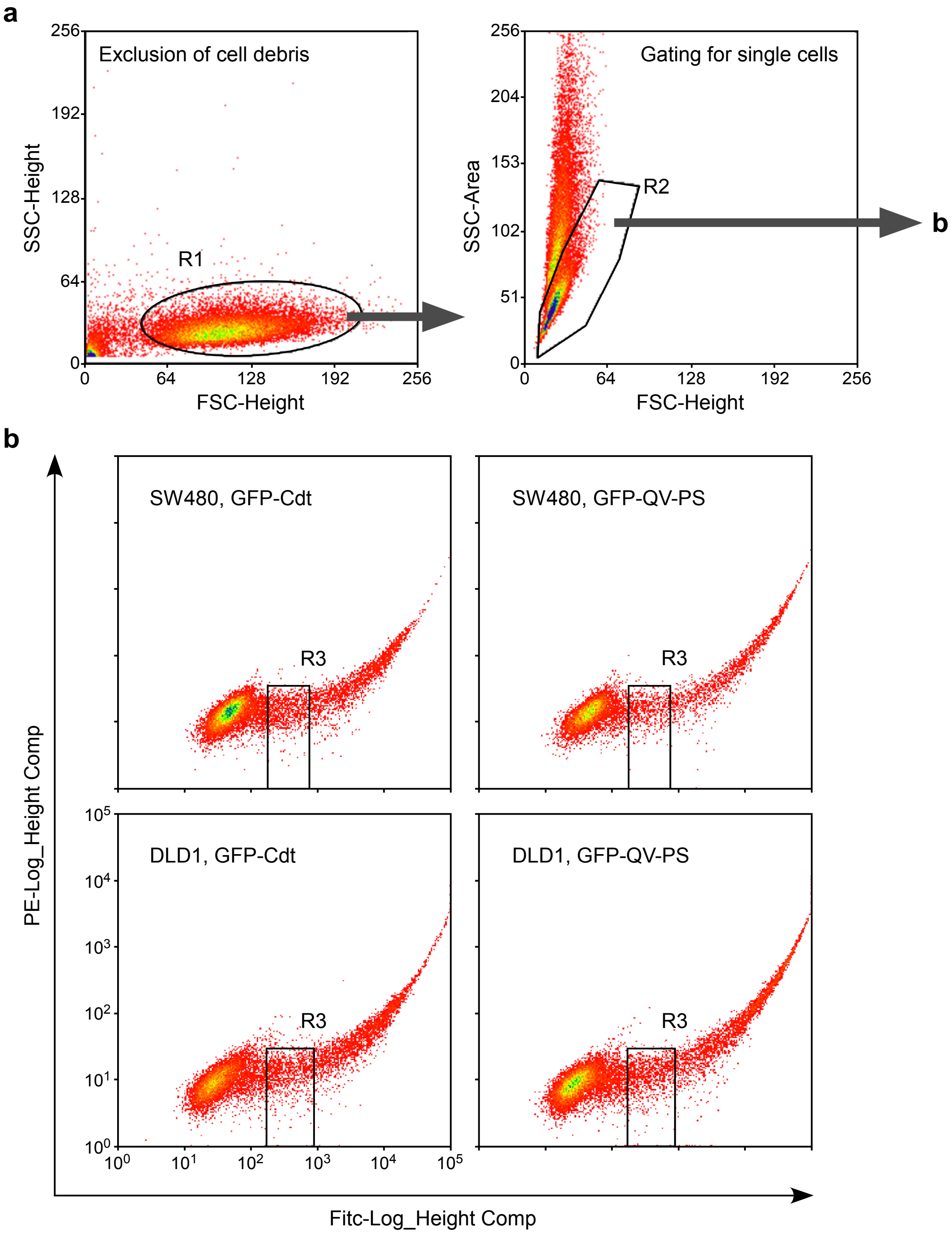

Supplementary Fig. 8 Cell sorting for the MTT assay shown in figure 4e. **a** Gating strategy: First, cells were sorted based on forward scatter (FSC) and side scatter (SSC) heights (R1) to exclude cell debris bevor excluding cell doublets based on forward scatter height and side scatter area to sort single cells (R2). The example shows sorting of DLD1 cells which tend to form doublets to a certain degree. **b** Sorting of SW480 cells (upper row) or DLD1 cells (lower row) expressing indicated GFP-tagged proteins at low levels. Identical sorting gates (R3) for conductin (Cdt) and the QV-PS mutant ensured that cells expressing comparable amounts of GFP-Cdt or GFP-QV-PS were analyzed for proliferation.

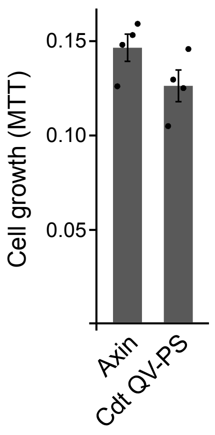

Supplementary Fig. 9 Similar proliferation of SW480 cells expressing axin or conductin QV-PS. MTT absorbance reflecting the number of viable SW480 cells expressing GFP-tagged axin or conductin QV-PS (Cdt QV-PS) 96 h after sorting and seeding. Cells expressing indicated GFP-tagged proteins at similar levels were sorted according to the gating strategy depicted in Supplementary Figure 8. One out of three representative experiments is shown. Results are mean +/- SEM of four replicates (n=4). Source data are provided as a Source Data file.

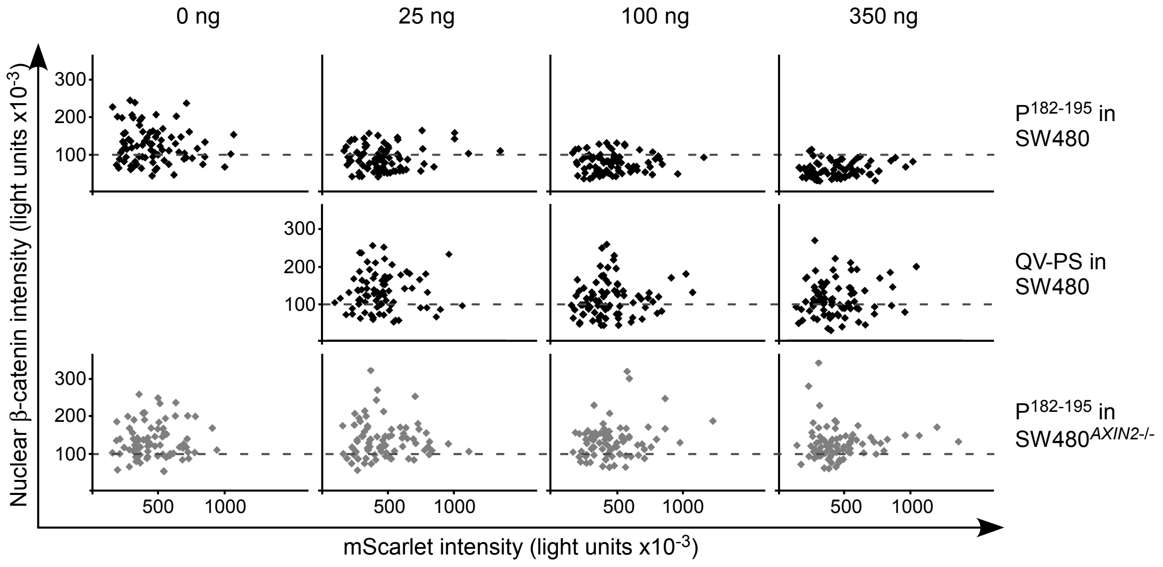

Supplementary Fig. 10 P182-195 induces β-catenin degradation via axin2. Dot plots of nuclear β-catenin intensities plotted against the corresponding mScarlet intensities of individual cells analyzed in figure 6b and c to show distribution of the data sets. Source data are provided as a Source Data file.

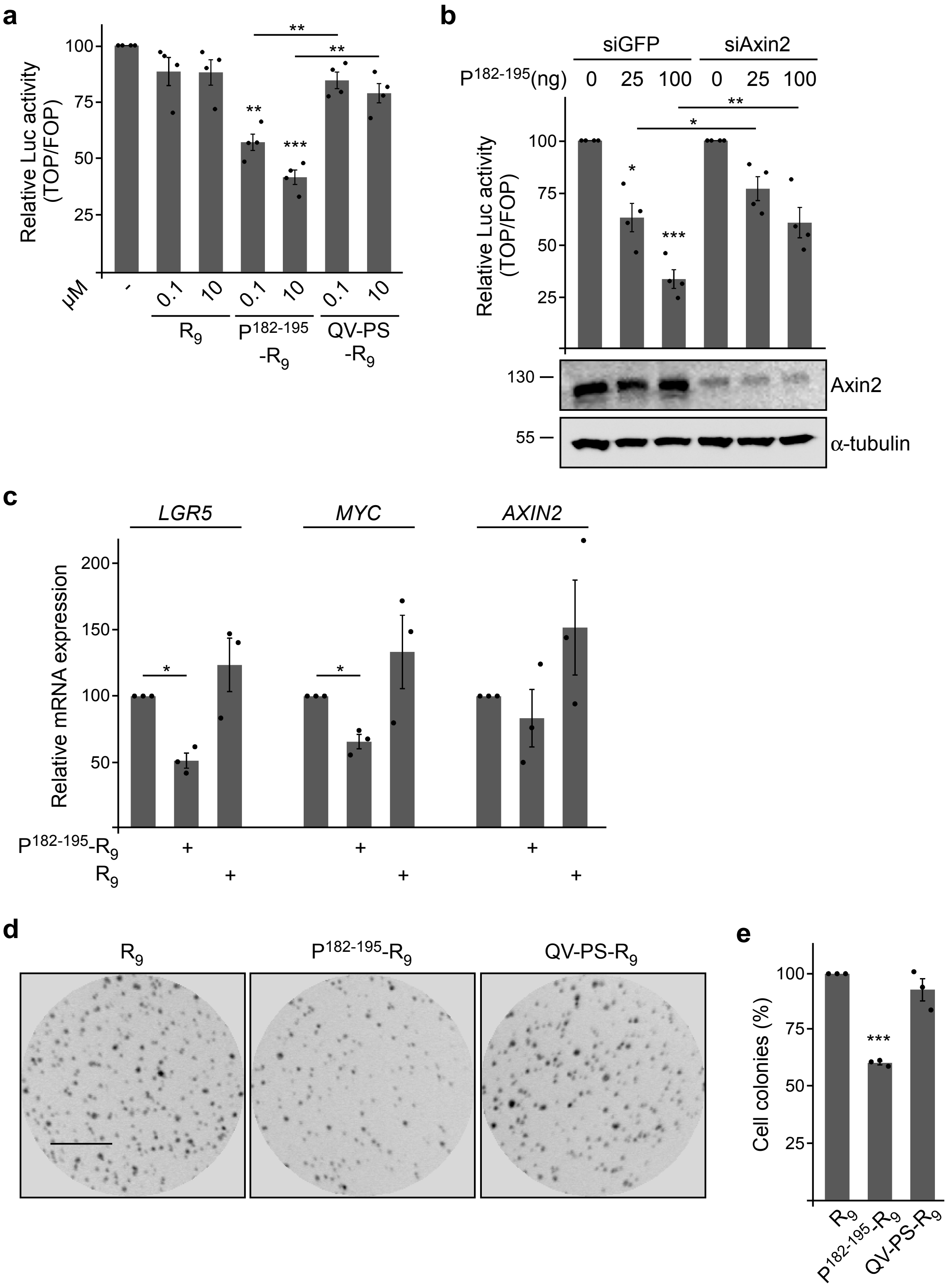

a
Relative Luc activity (TOP/FOP)
100
75
50
25
μM
-
0.1
10
R9
P182-195 -R9
QV-PS -R9
**
***
b
siGFP
siAxin2
P182-195(ng) 0 25 100 0 25 100
Relative Luc activity (TOP/FOP)
*
**
***
130 —
Axin2
55 —
α-tubulin
c
LGR5
MYC
AXIN2
Relative mRNA expression
200
150
100
50
P182-195-R9
R9
+
d
R9
P182-195-R9
QV-PS-R9
e
Cell colonies (%)
R9
P182-195-R9
QV-PS-R9

Supplementary Fig. 11 $P^{182\text{-}195}$ inhibits Wnt signaling and blocks growth of DLD1 colorectal cancer cells. **a, b** Luciferase activity (TOP/FOP) in DLD1 cells which were untreated, or treated with indicated amounts of a 9x arginine control peptide (R_9), a $P^{182\text{-}195}$-R_9 fusion or the QV-PS mutated analog (QV-PS-R_9) for 48 h (**a**), in DLD1 cells which were transfected with $P^{182\text{-}195}$ together with siRNA against GFP (control) or against axin2 (**b**). Results are mean +/- SEM (n=4). Western blot below b shows efficient axin2 knockdown. Given nanograms (ng) of $P^{182\text{-}195}$ refer to the transfection of a 12-well. **c** Relative mRNA expression of the β-catenin target genes *LGR5*, *MYC* and *AXIN2* normalized to *GAPDH* in DLD1 cells which were untreated or treated for 48 h with 10 µM of indicated peptides. Results are mean +/- SEM (n=3). **d** Cell colonies grown for 96 h from DLD1 cells which were treated with 10 µM of indicated synthetic peptides. Cells were stained by ethidium bromide incorporation and visualized with UV light. Scale bar: 0.5 cm. **e** Automated quantification of colony numbers from three independent experiments as in d. Results are mean +/- SEM (n=3). *$p<0.05$, **$p<0.01$, ***$p<0.001$ (Student's *t*-test). Source data are provided as a Source Data file.

Supplementary References

1 Fernandez-Escamilla, A. M., Rousseau, F., Schymkowitz, J. & Serrano, L. Prediction of sequence-dependent and mutational effects on the aggregation of peptides and proteins. *Nat Biotechnol* **22**, 1302-1306, doi:10.1038/nbt1012 (2004).

2 Sievers, F. *et al.* Fast, scalable generation of high-quality protein multiple sequence alignments using Clustal Omega. *Molecular systems biology* **7**, 539, doi:10.1038/msb.2011.75 (2011).

3 Petersen, B., Petersen, T. N., Andersen, P., Nielsen, M. & Lundegaard, C. A generic method for assignment of reliability scores applied to solvent accessibility predictions. *BMC structural biology* **9**, 51, doi:10.1186/1472-6807-9-51 (2009).
