# Supplementary material for: An aggregon in conductin/axin2 regulates Wnt/β-catenin signaling and holds potential for cancer therapy
Source: Nat Commun. 2019 Sep 18;10:4251. doi: 10.1038/s41467-019-12203-8 (PMC6751202; doi:10.1038/s41467-019-12203-8)
Supplement: Supplementary file 3 — Reporting Summary [file 41467_2019_12203_MOESM3_ESM.pdf]

nature research

Corresponding author(s): Dominic B. Bernkopf

Last updated by author(s): 15.08.2019

Reporting Summary

Nature Research wishes to improve the reproducibility of the work that we publish. This form provides structure for consistency and transparency in reporting. For further information on Nature Research policies, see Authors & Referees and the Editorial Policy Checklist.

Statistics

For all statistical analyses, confirm that the following items are present in the figure legend, table legend, main text, or Methods section.

n/a	Confirmed	
☐	☒	The exact sample size (*n*) for each experimental group/condition, given as a discrete number and unit of measurement
☐	☒	A statement on whether measurements were taken from distinct samples or whether the same sample was measured repeatedly
☐	☒	The statistical test(s) used AND whether they are one- or two-sided<br>*Only common tests should be described solely by name; describe more complex techniques in the Methods section.*
☒	☐	A description of all covariates tested
☐	☒	A description of any assumptions or corrections, such as tests of normality and adjustment for multiple comparisons
☐	☒	A full description of the statistical parameters including central tendency (e.g. means) or other basic estimates (e.g. regression coefficient) AND variation (e.g. standard deviation) or associated estimates of uncertainty (e.g. confidence intervals)
☒	☐	For null hypothesis testing, the test statistic (e.g. *F*, *t*, *r*) with confidence intervals, effect sizes, degrees of freedom and *P* value noted<br>*Give P values as exact values whenever suitable.*
☒	☐	For Bayesian analysis, information on the choice of priors and Markov chain Monte Carlo settings
☒	☐	For hierarchical and complex designs, identification of the appropriate level for tests and full reporting of outcomes
☒	☐	Estimates of effect sizes (e.g. Cohen's *d*, Pearson's *r*), indicating how they were calculated

Our web collection on statistics for biologists contains articles on many of the points above.

Software and code

Policy information about availability of computer code

Data collection	Bio-Rad CFX Manager 3.0 (qRT-PCR), E.A.S.Y. Win32 4.00.233 (cell colony images), ImageReader LAS-3000 2.0 (WB), MetaMorph 6.2r4 (IF), MikroWin 2000 4.29 (reporter assay), SoftMax Pro v.5.4.1 (reporter assay, MTT assay), SWISS-MODEL (RGS structure model)
Data analysis	Aida Image Analyzer v.3.52 (WB, 2D densitometry), Clustal Omega 1.2.4 (alignments), MetaMorph 6.2r4 (IF, cell colonies), Microsoft Excel 14.0.7232.5000, NetSurfP 1.1 (protein surface accessibility), TANGO 2.2 (aggregation propensity)

For manuscripts utilizing custom algorithms or software that are central to the research but not yet described in published literature, software must be made available to editors/reviewers. We strongly encourage code deposition in a community repository (e.g. GitHub). See the Nature Research guidelines for submitting code & software for further information.

Data

Policy information about availability of data

All manuscripts must include a data availability statement. This statement should provide the following information, where applicable:

- Accession codes, unique identifiers, or web links for publicly available datasets
- A list of figures that have associated raw data
- A description of any restrictions on data availability

Data supporting the findings of this manuscript are available from the corresponding author upon reasonable request. The source data underlying Figs. 1 E, 2 D, G, 3 A-D, 4 B-E, 5 C, D, F-M, 6 B-F, 7 A-H, J, L, M, S4 C-E, S7, S9, S10 and S11 A-C, E are provided as a Source Data file.

Field-specific reporting

Please select the one below that is the best fit for your research. If you are not sure, read the appropriate sections before making your selection.

☒ Life sciences ☐ Behavioural & social sciences ☐ Ecological, evolutionary & environmental sciences

For a reference copy of the document with all sections, see nature.com/documents/nr-reporting-summary-flat.pdf

Life sciences study design

All studies must disclose on these points even when the disclosure is negative.

Sample size	Sample sizes were chosen according to previous experiences and suited to obtain statistical significant results.
Data exclusions	No data of decent experimental quality was excluded.
Replication	All experimental findings were reproduced in form of at least three independent biological replicates.
Randomization	Samples, i.e. seeded cells, were allocated randomly into experimental groups.
Blinding	For most experiments blinding was unnecessary due to automated objektive readout. When required, e.g. when assessing less pronounced effects at the microscope, the investigator was blinded with respect to the samples.

Reporting for specific materials, systems and methods

We require information from authors about some types of materials, experimental systems and methods used in many studies. Here, indicate whether each material, system or method listed is relevant to your study. If you are not sure if a list item applies to your research, read the appropriate section before selecting a response.

Materials & experimental systems

n/a	Involved in the study	
☐	☒	Antibodies
☐	☒	Eukaryotic cell lines
☒	☐	Palaeontology
☒	☐	Animals and other organisms
☒	☐	Human research participants
☒	☐	Clinical data

Methods

n/a	Involved in the study	
☒	☐	ChIP-seq
☒	☐	Flow cytometry
☒	☐	MRI-based neuroimaging

Antibodies

Antibodies used

anti alpha-tubulin (Serotec, MCA77G, YL1/2)
anti APC (Abcam, ab58, Ali 12-28)
anti Axin2 (CellSignaling, 2151S, 76G6)
anti beta-actin (Sigma-Aldrich, A5441, AC-15)
anti beta-catenin (Santa Cruz Biotechnologies, sc-7963, E-5)
anti Flag (Sigma-Aldrich, F7425)
anti GFP (Roche, 11814460001, 7.1, 13.1)
anti GST (CellSignaling, 2624S, 26H1)
anti HA (Sigma-Aldrich, H6908)

anti mouse-Cy2 (Jackson ImmunoResearch, 115-225-146)
anti mouse-Cy3 (Jackson ImmunoResearch, 115-165-146)
anti mouse-HRP (Jackson ImmunoResearch, 115-035-146)
anti rabbit-Cy2 (Jackson ImmunoResearch, 111-225-144)
anti rabbit-Cy3 (Jackson ImmunoResearch, 111-165-144)
anti rabbit-HRP (Jackson ImmunoResearch, 111-035-144)
anti rat-HRP (Jackson ImmunoResearch, 112-035-143)

Validation

All primary antibodies are commercially available, and valid for the described applications according to statements by the supplier and references at the suppliers homepage. In addition, bands of correct molecular weight were detected (WB), signal for anti tag antibodies was absent without expression of tagged proteins, and, when available, signal loss in knockout cells or signal reduction after knockdown were verified in the lab.

Eukaryotic cell lines

Policy information about cell lines

Cell line source(s)	Source of all cell lines: ATCC.
Authentication	During culturing cells were authenticated based on their morphology and indicative features such as e.g. truncated APC in SW480 and DLD1 cells.
Mycoplasma contamination	All cell lines were tested negative for mycoplasma contamination.
Commonly misidentified lines (See ICLAC register)	None of the used cell lines is commonly misidentified according to the ICLAC register.
